# Improved oncological outcome after radical prostatectomy in patients staged with ^68^Ga-PSMA-11 PET: a single-center retrospective cohort comparison

**DOI:** 10.1007/s00259-020-05058-5

**Published:** 2020-10-19

**Authors:** Daniela A. Ferraro, Fabienne Lehner, Anton S. Becker, Benedikt Kranzbühler, Ken Kudura, Iliana Mebert, Michael Messerli, Thomas Hermanns, Daniel Eberli, Irene A. Burger

**Affiliations:** 1 Department of Nuclear Medicine, University Hospital Zurich, University of Zurich, Rämistrasse 100, 8091 Zürich, Switzerland; 2 Department of Urology, University Hospital Zurich, University of Zurich, Zurich, Switzerland; 3 Institute of Diagnostic and Interventional Radiology, University Hospital Zurich, University of Zurich, Zurich, Switzerland; 4 Department of Radiology, Memorial Sloan Kettering Cancer Center, New York City, NY USA; 5 Department of Nuclear Medicine, Kantonsspital Baden, Baden, Switzerland

**Keywords:** Oncological outcome, PET/CT, PET/MR, PSMA PET, Postsurgical outcome, Radical prostatectomy

## Abstract

**Background:**

Positron emission tomography (PET) targeting the prostate-specific membrane antigen (PSMA) has superior sensitivity over conventional imaging (CI) to stage prostate cancer (PCa) and therefore is increasingly used in staging to stratify patients before radical therapy. Whether this improved diagnostic accuracy translates into improved outcome after radical prostatectomy (RPE) has not yet been shown. Therefore, the aim of this study was to compare the oncological outcome after RPE between patients that underwent preoperative staging with CI or PSMA-PET for intermediate and high-risk PCa.

**Methods:**

We retrospectively selected all patients that underwent RPE for intermediate- or high-risk PCa at our institution before PSMA-PET introduction (between March 2014 and September 2016) and compared the oncologic outcome of patients staged with PSMA-PET (between October 2016 and October 2018). Oncological pre-surgical risk parameters (age, PSA, D’Amico score, biopsy-ISUP, and cT stage) were compared between the groups. Oncological outcome was determined as PSA persistence, nerve-sparing rate, and surgical margin status. Wilcoxon rank-sum, Fisher’s, and chi-square tests where used for statistical testing.

**Results:**

One hundred five patients were included, 53 in the CI group and 52 in the PSMA-group. Patients in the PSMA group had higher ISUP grade (*p* < 0.001) and D’Amico score (*p* < 0.05). The rate of free surgical margins and PSA persistence after RPE was 64% and 17% for the CI and 77% and 6% for the PSMA group (*p* = 0.15 and 0.13, respectively). Subgroup analysis with high-risk patients revealed PSA persistence in 7% (3/44) in the PSMA group and 25% (7/28) in the CI group (*p* = 0.04). Limitations include the retrospective design and choline-PET for some patients in the CI group.

**Conclusion:**

Immediate outcome after RPE was not worse in the PSMA group compared with the CI group, despite a higher-risk cohort. In a comparison of only high-risk patients, PSMA-PET staging was associated with a significantly lower rate of postsurgical PSA persistence.

## Introduction

Several single-center studies suggested an improved sensitivity of positron emission tomography (PET) targeting the prostate-specific membran antigen (PSMA) for staging intermediate- to high-risk prostate cancer (PCa) [1–4]. Recently this benefit of PSMA staging has been confirmed in a randomized prospective multicenter trial (proPSMA), which revealed an improved sensitivity for nodal and distant metastasis for PSMA-PET compared with conventional imaging (CI) [5]. Furthermore, in a direct comparison with magnetic ressonance imaging (MRI), PSMA-PET/MRI has a higher reported sensitivitiy for extracapsular extension and seminal vesicle invasion, of up to 94% and 95% [6–8].

Six publications investigating the impact of PSMA-PET on management were published between 2018 and 2019 including a total of 396 patients, showing a range of management change from 21 to 52% [3, 8–12]. PSMA-PET was considered useful for improved patient selection for focal therapies, or to change therapy modalities based on the PET findings as shown also in a study from our group that found a change in management in 27% of patients based on the staging PSMA-PET [10, 13]. Furthermore, some preliminary results suggest that PSMA-PET could further improve the selection of patients for pelvic nodal dissection (pLND), considering that up to 47% of the detected lymph node metastasis are not found within the regular pLND areas [14]. The accuracy of PSMA-PET to select patients for pLND is as high as the existing nomograms currently used, and there are first results showing that a combination of quantitative assessment of PSMA in the primary tumor with visual analysis of the nodes on PET could improve selection for pLND even further [7, 15].

In the light of these data, it could be expected that the higher sensitivity of PSMA-PET for metastasis detection leads to a better selection of patients for radical prostatectomy (RPE). After surgery, immediate oncological success of the treatment can be measured by the rate of negative surgical margins and an undetectable PSA (< 0.1 ng/ml) after surgery. PSA persistence 6 weeks after RPE is associated with worse oncological outcome [16].

However, the surgical outcome after RPE for patients that underwent PSMA-PET for staging PCa has not yet been investigated. Therefore, we aimed to compare the immediate outcome after RPE between patients with intermediate and high-risk PCa, who underwent the same pre-surgical and surgical procedures in a single center, staged with either CI or ^68^Ga-PSMA-11 PET/CT or PET/MR.

## Patients and methods

### Study population

**Fig. 1:**
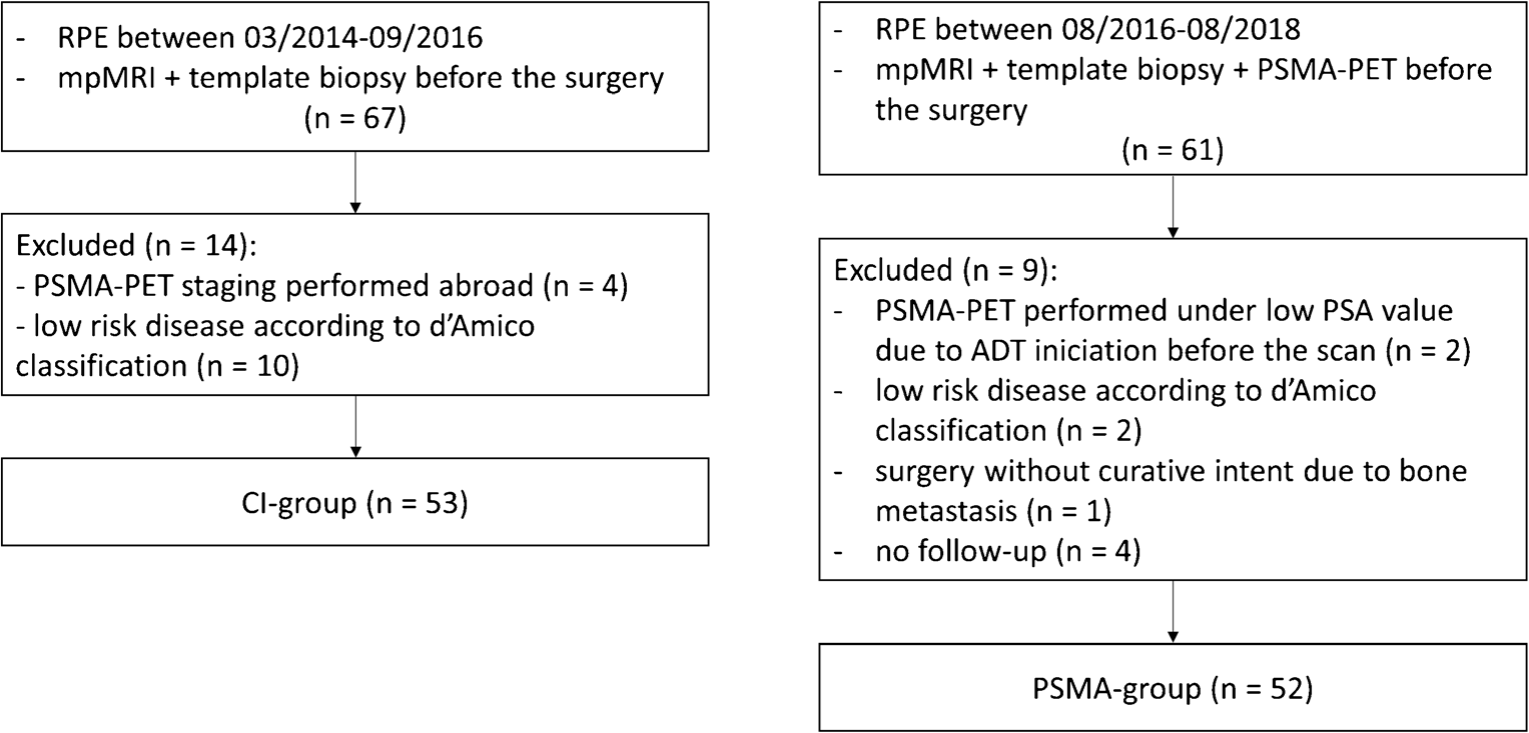
Flow diagram of inclusion of patients. ADT = androgen deprivation therapy; CI = conventional imaging; mpMRI = multiparametric magnetic resonance imaging; PSA = prostate-specific antigen; PSMA-PET = positron emission tomography with prostate-specific membrane antigen; RPE = radical prostatectomy

This retrospective study included patients with intermediate- or high-risk PCa who underwent RPE between March 2014 and October 2018 and had a preoperative multiparametric MRI (mpMRI) followed by a template saturation biopsy (20 or more cores). All consecutive patients surgically treated between March 2014 and September 2016 with conventional staging, including choline PET/CT, contrast enhanced CT, bone scan, or abdominal MRI, compose the CI group. PSMA-PET was introduced in our institution in April 2016. Therefore, patients surgically treated between October 2016 and October 2018 were staged with PSMA-PET and compose the PSMA group (Fig. 1). Between March 2014 and October 2018, there was no change in the standard surgery procedures for RPE and no change in the operating team. Relevant clinical and pathological information was collected from the patients’ medical charts. The two groups were compared regarding initial risk according to both D’Amico risk classification and the recently published criteria in the proPSMA trial, as well as age, TNM stage, PSA level, and both biopsy and RPE specimen–derived ISUP grade groups. Most of the PSMA cohort (49/52) was analyzed in the recently published outcome paper on change in management [13]. The local ethics committee approved the study protocol, and all patients gave a general written informed consent for retrospective use of their data (BASEC Nr. 2018-01284).

### Imaging techniques and analysis

Patients underwent a clinical routine ^68^Ga-PSMA-11 PET/MRI (SIGNA PET/MR, GE Healthcare, Waukesha, WI, USA) or ^68^Ga-PSMA-11 PET/CT (Discovery VCT 690 or Discovery MI PET/CT, GE Healthcare, Waukesha, WI, USA). Images were acquired 60 min after injection of ^68^Ga-PSMA-11 (mean dose ± standard deviation (SD), 131 ± 19 MBq, range 81–160 MBq). To reduce activity in the urinary system, furosemide was injected intravenously 30 min prior to the tracer injection (0.13 mg/kg) and patients were asked to void prior to the scan. The institutional protocol was in agreement with the EANM and SNMMI procedure guidelines [17]. Studies were reported in clinical routine by experienced dual board-certified radiologists and nuclear medicine physicians, avoiding known PSMA-positive pitfalls [18, 19]. More details regarding imaging protocol and analysis are given in the supplements.

### Radical prostatectomy and lymphadenectomy

All surgical procedures were performed in form of a robot-assisted transperitoneal laparoscopic RPE with bilateral extended pLND using the four-arm Da Vinci S system (Intuitive Surgical, Inc., USA) by experienced urologists at our institution as described earlier [20]. Extended pLND is a standard procedure for all intermediate- and high-risk PCa patients that undergo RPE in our hospital irrespective of the result of PSMA-PET imaging and included the external iliac, obturator, and internal iliac (hypogastric) lymph nodes with an upper resection boundary defined by the crossing of the ureter over the common iliac artery. If PSMA-PET revealed nodal metastases in the pelvis outside the template (e.g., common iliac nodes), these nodes were also removed if technically possible. Nerve-sparing was performed if imaging showed no cancer close to the bundle region, capsular involvement, or suspicion for extracapsular extension. In all cases with nerve-sparing, frozen section was used during surgery and the neurovascular bundle removed, if the prostate surface showed cancer.

### Outcome definitions

Surgical outcome was assessed by the rate of nerve-sparing in each group (patient-wise and side-wise), surgical margins status (R0 or R1), PSA persistence after surgery (defined as PSA level ≥ 0.1 ng/ml measured 6 weeks after surgery) [16]. Information was collected from patients’ charts.

### Statistical analysis

Statistical analysis was performed with R version 3.6.3. Descriptive statistics were expressed as median and interquartile range (IQR) (25th and 75th percentiles (Q1-Q3)), as well as counts and percentages. To compare different groups, the following statistical tests were performed: Wilcoxon rank-sum test, Fisher’s exact test, and chi-square test of independence, where appropriate. All tests were two-tailed. A *p* value < 0.05 was considered indicative of significant differences. In the subgroup analysis, we did not correct for multiple comparisons due to the exploratory nature of the analysis.

## Results

One hundred five patients were included, 53 in the CI group and 52 in the PSMA group (Table 1). Information about imaging staging for distant disease in the CI group is shown in Table 2; of note, 24 (45%) patients underwent choline PET; 21 of them had high-risk disease representing 75% of the high-risk patients (21 of 28). In the PSMA group, 42 patients underwent ^68^Ga-PSMA-11 PET/MRI and ten patients had PET/CT. No patient had findings of M1b or M1c disease on CI or PSMA-PET. Two patients with PSMA-positive common iliac nodes were still considered as pelvic disease and referred for RPE.

**Table 1:** Characteristics of the patients

	CI group (*n* = 53)	PSMA group (*n* = 52)	*p* value
Age median (IQR)	65 (62–69)	65 (61–69)	> 0.9
PSA median (IQR)	8 (6–13)	9 (5–14)	0.8
< 10	32 (60.5%)	28 (54%)	
10–20	12 (22.5%)	19 (36.5%)	
> 20	9 (17%)	5 (9.5%)	
cT stadium *n* (%)			0.11
T1c	46 (87%)	37 (71%)	
T2a	2 (3.5%)	7 (13.5%)	
T2b	1 (2%)	0	
T2c	3 (5.5%)	7 (13.5%)	
T3	1 (2%)	1 (2%)	
ISUP grade group on biopsy *n* (%)			< 0.001
2	17 (32%)	4 (7.5%)	
3	13 (24.5%)	8 (15.5%)	
4	8 (15%)	25 (48%)	
5	15 (28.5%)	15 (29%)	
D’Amico risk group *n* (%)			< 0.001
Intermediate	25 (47%)	8 (15.5%)	
High	28 (53%)	44 (84.5%)	
Risk according to proPSMA trial* *n* (%)			0.002
Low	12 (22.5%)	1 (2%)	
Intermediate	3 (6%)	2 (4%)	
High	38 (71.5%)	49 (94%)	
ISUP grade group on prostatectomy specimen *n* (%)			0.008
1	0	1 (2%)	
2	18 (34%)	5 (9.5%)	
3	22 (42%)	23 (44%)	
4	4 (7.5%)	12 (23%)	
5	9 (17%)	11 (21%)	

*According to criteria used in the proPSMA trial (high-risk: ISUP ≥ 3, PSA ≥ 20 ng/ml, or clinical T3 or more) [5], low risk was considered as ISUP ≤ 2, PSA < 10, and cT1c

**Table 2:** Imaging modalities for staging of distant disease before radical prostatectomy in the conventional imaging group (*n* = 53), according to disease risk using D’Amico risk score

	Intermediate risk	High risk	Total
Choline PET	3	21	24
Bone scan and/or CT	1	4	5
No imaging	20*	0	20
Data not available	1	3	4
Total	25	28	53

*13 patients had ISUP grade 2 on biopsy, and therefore, no imaging was indicated to investigate distant metastasis according to the updated European Association of Urology guideline. The remaining 7 patients had ISUP grade 3 on biopsy, T1c disease and PSA < 10 ng/ml

*CT* computed tomography

### Comparison of patients and disease characteristics between the groups

**Fig. 2:**
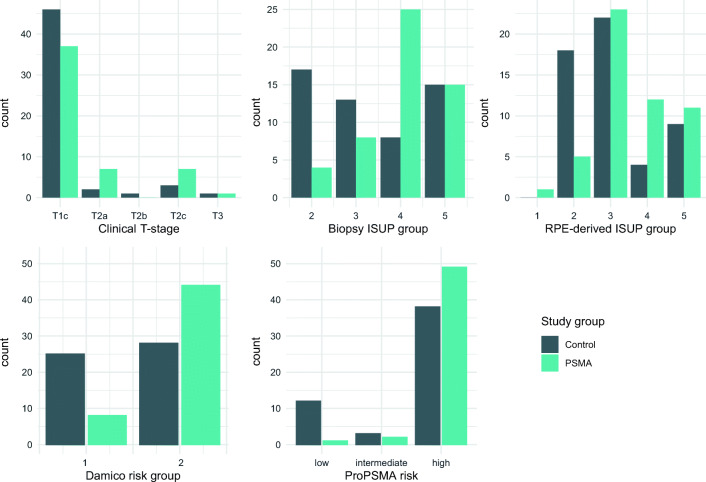
Comparison of oncological pre-surgical risk parameters between the CI and PSMA groups. Data show a relatively similar distribution between the two groups for clinical T stadium but a higher risk cohort in the PSMA group when compared with the CI group according to ISUP grade groups (on biopsy and on radical prostatectomy (RPE) specimen) and both D’Amico risk classification and criteria used in the proPSMA trial

Characteristics of both groups are shown in Table 1 and Fig. 2. There was no difference between PSMA and CI groups regarding age, initial PSA level, and clinical T-stage. However, in the PSMA group significantly more patients had high-risk disease compared with the CI group, mainly due to higher biopsy ISUP grade (*p* < 0.001). In the CI group, the most frequent tumor biopsy ISUP grade was 2 (32%, 17/53) and only 53% (28/53) of the patients had high-risk disease according to D’Amico score, while in the PSMA group, around half of the patients (48%, 25/52) presented ISUP grade 4 tumors and 84.5% (44/52) had high-risk disease. After RPE, pathological tumor grade confirmed the difference between the cohorts, with 24.5% of patients presenting tumors grade 4 or 5 in the CI group and 44% in the PSMA group. The number of resected lymph nodes at extended pLND was similar for both groups, with median of 21 nodes in both (mean 20.4 nodes, IQR 15–24 in the CI group and mean 22.6 nodes, IQR 17–26 in the PSMA group). pN1 disease was found in 7 patients (13.5%) in the CI group and in nine (17%) in the PSMA group (*p* = 0.8), with a mean of metastatic nodes of 3 (mean 1.0, range 1–11) and 2.5 (mean 2.0, range 1–7), respectively. Thirty-three patients had pT2 and 20 pT3 disease in the CI group, compared with 35 and 17 in the PSMA group, respectively. Among the patients with pT2 disease, the rate of R1 margins was 15% (5/33) in the CI group and 8**.**6% (3/35) in the PSMA group.

### Comparison of outcome between the groups

**Fig. 3:**
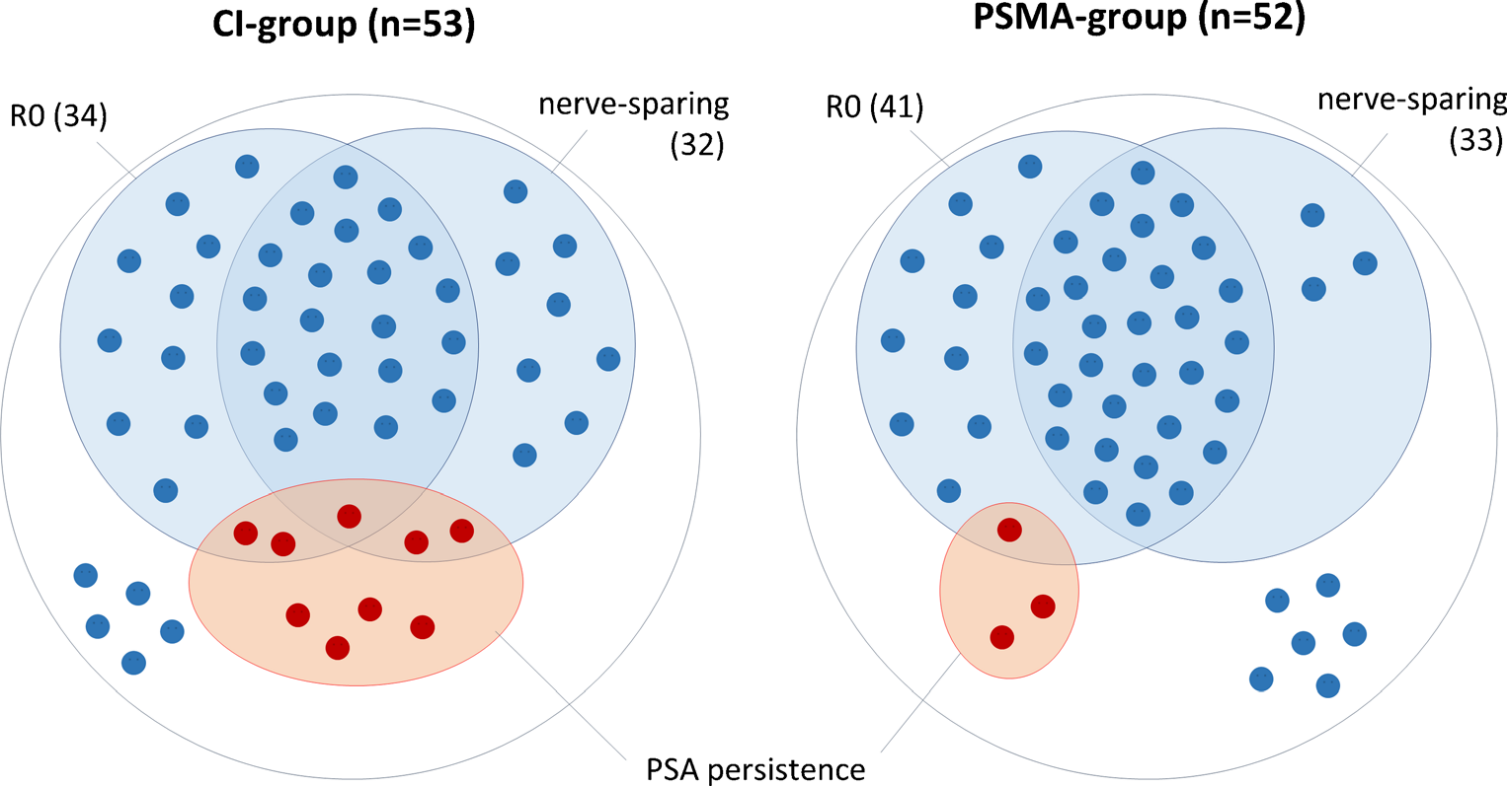
Comparison of outcome between the CI and PSMA groups. Venn diagrams showing the distribution of the 105 patients (represented as dots) in three sets: those who had nerve-sparing, those who had free surgical margins (R0), and those who had PSA persistence after radical prostatectomy (RPE). In the CI group, the intersection between the sets of patients who had nerve-sparing and R0 has fewer patients (21) than in the PSMA group (30). In the CI group, three patients with nerve-sparing had PSA persistence, against none in the PSMA group. Patients who are not placed inside any of the three sets are those who did not have nerve-sparing, had positive margins (R1), and had undetectable PSA after RPE (five patients in the CI group and six in the PSMA group)

Results of this section are presented in Table 3 and illustrated in Fig. 3.

**Table 3:** Comparison of outcome between the PSMA and CI groups

	All patients (*n* = 105)			High-risk subgroup* (*n* = 72)		
	CI group (*n* = 53)	PSMA group (*n* = 52)	*p* value	CI group (*n* = 28)	PSMA group (*n* = 44)	*p* value
Nerve-sparing *n* (%)	32 (60%)	33 (63%)	> 0.9	13 (46%)	29 (66%)	0.2
Surgical margins *n* (%)			0.15			0.12
R0	34 (64%)	41 (79%)		16 (57%)	34 (77%)	
R1	19 (36%)	11 (21%)		12 (43%)	10 (23%)	
PSA status after RPE *n* (%)			0.13			0.04
< 0.1 ng/ml	44 (83%)	49 (94%)		21 (75%)	41 (93%)	
≥ 0.1 ng/ml	9 (17%)	3 (6%)		7 (25%)	3 (7%)	

*According to D’Amico score

PSA: prostate-specific antigen; RPE: radical prostatectomy

**Fig. 4:**
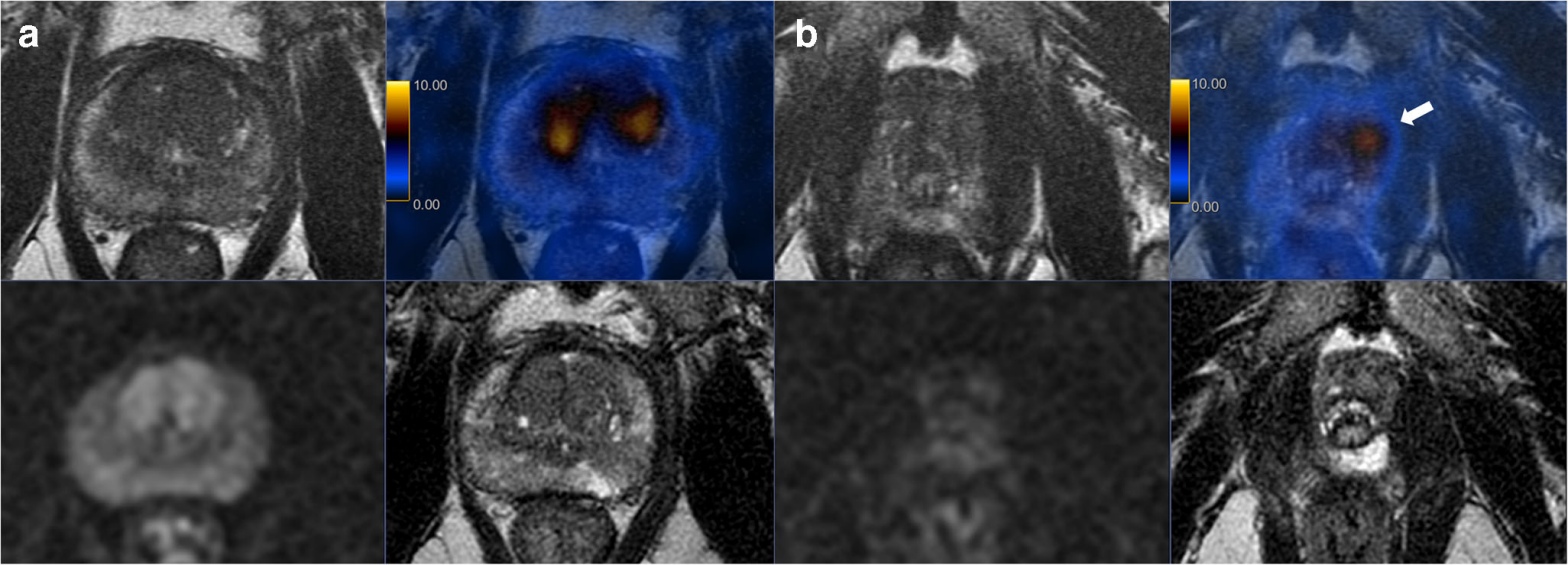
Extracapsular extension shown on ^68^Ga-PSMA-11 positron emission tomography/magnetic resonance imaging (^68^Ga-PSMA-11 PET/MRI). Staging prostate magnetic resonance imaging (bottom) and ^68^Ga-PSMA-11 PET/MRI (top) of a 75-year-old patient with a T1c tumor, Gleason score 4 + 3 = 7 on biopsy, and initial PSA level of 16 ng/ml. Imaging shows a lesion involving the transition zone of the prostatic base **(A)** and the anterior apex **(B)**. MRI (diffusion-weighted images and T2-weighted fast recovery fast spin-echo sequence (FRFSE)) shows a bilateral PIRADS 3 lesion in the peripheral zone without suspicion of extracapsular extension. ^68^Ga-PSMA-11 PET/MRI (FRFSE and fused PET/MRI) shows increased bilateral PSMA uptake, suggestive of significant cancer with suspicion of extracapsular extension on the left side (arrow). Surgery was performed without nerve spearing and histopathology confirmed pT3a disease

In a patient-based analysis, the rate of nerve-sparing was similar between the groups, with 60% (32/53) in the CI group versus 63% (33/52) in the PSMA group (*p* > 0.9). However, in the CI group, bilateral nerve-sparing was more often performed (56%, 18/32) than in the PSMA group (30%, 10/33). Among patients with any nerve-sparing, the rate of negative surgical margins was 69% (22/32) in the CI-group and 91% (30/33) for the PSMA-group. Of note, no patient in the PSMA-group who had nerve-sparing had PSA persistence (Fig. 4), against 3 patients (9%) in the CI group (two with R1 and one with R0 resection). For high-risk patients according to D’Amico risk score, the patient-based rate of nerve-sparing in the PSMA group was 66% and 46% in the CI group (*p* = 0.2).

**Fig. 5:**
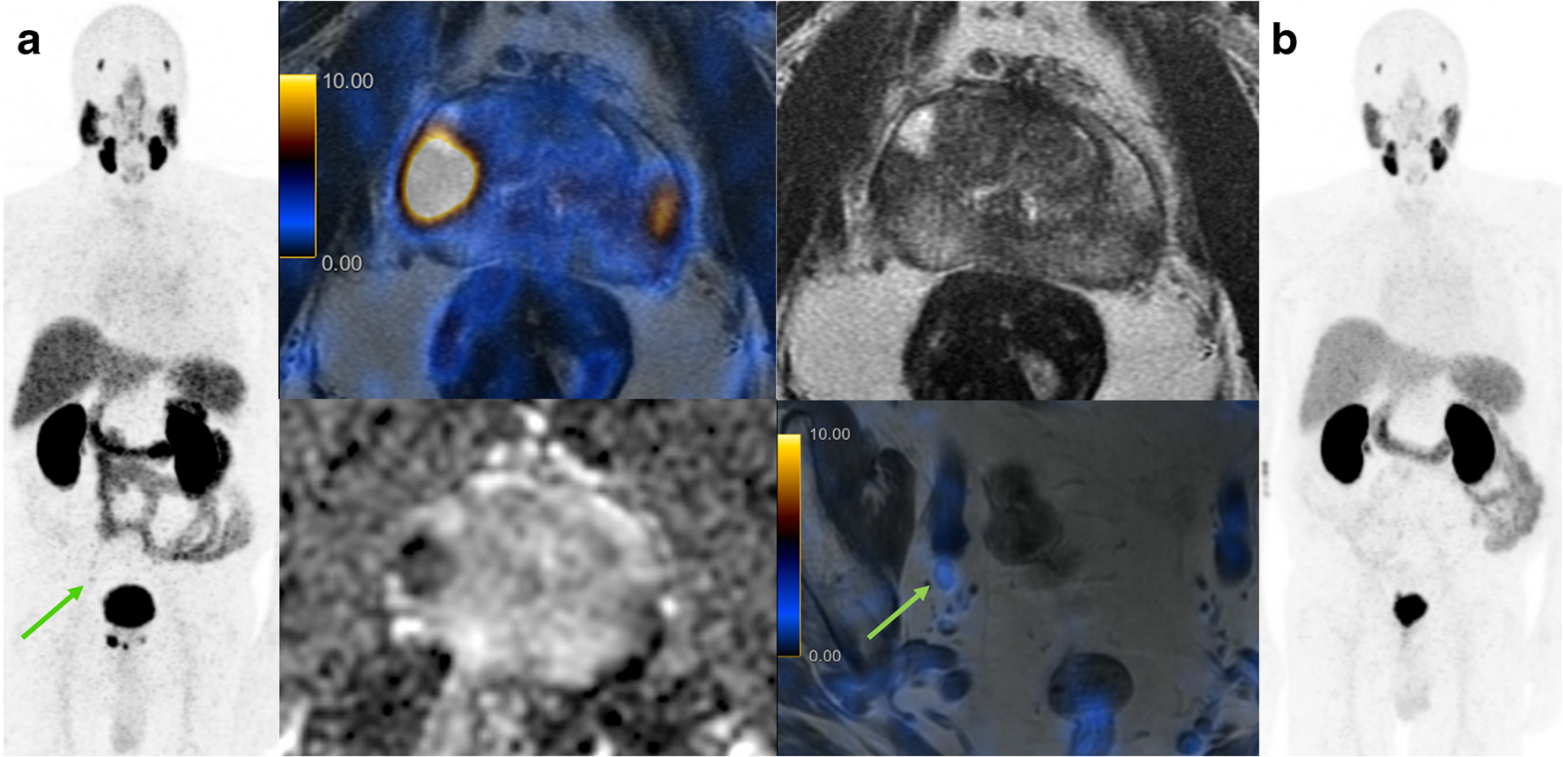
^68^Ga-PSMA-11 positron emission tomography/magnetic resonance imaging (PET/MRI) from the only patient who had postsurgical prostate-specific antigen (PSA) persistence with free surgical margins in the PSMA group. ^68^Ga-PSMA-11 PET/MRI studies in different moments of a 73-year-old patient with a T1c tumor, Gleason score 4 + 5 = 9 on biopsy, and initial PSA level of 25.6 ng/ml. **(A)** Staging PET showed the primary tumor with a high PSMA uptake (SUV_max_ 25) and a single PSMA-positive iliac internal lymph node (arrows). The patient underwent radical prostatectomy with extended pelvic lymph node dissection (R0, pN1, 1/25) but had PSA persistence (0.82 ng/ml). **(B)** Subsequent PET showed no suspicious lesion and the patient underwent salvage radiotherapy with a PSA drop to 0.35 ng/ml

Overall, the rate of negative surgical margins and undetectable PSA after RPE was slightly higher in the PSMA group (*p* = 0.15 and *p* = 0.13, respectively). For high-risk patients, the difference is accentuated: 57% of R0 and 75% of undetectable PSA after RPE in the CI group and 77% and 93% in the PSMA group (*p* = 0.12 and 0.04), respectively. Among patients with R0, the rate of PSA persistence was 8.8% (3/34) in the CI group and 2.4% (1/41) in the PSMA group (Fig. 5).

## Discussion

This is the first cohort comparison of oncological post-RPE outcome in patients that underwent either PSMA-PET or CI for staging. Our analysis revealed an improvement of surgical planning with a higher rate of unilateral nerve-sparing and a lower rate of positive surgical margins, most likely due to better preoperative identification of the location of the tumor using PSMA-PET imaging. Furthermore, the hypothesis that patient selection with PSMA-PET for RPE was superior compared with CI was confirmed with only one versus three patients that had PSA persistence despite R0 resection. Despite higher biopsy ISUP grades and fewer D’Amico intermediate-risk patients, the post RPE outcome regarding PSA persistence was non-inferior between both groups. Subgroup analysis between high-risk patients showed that the PSMA group had a significantly lower rate of PSA persistence 6 months after RPE compared with the CI group.

Our findings are supported by the increasing number of publications that show a higher accuracy for ^68^Ga-PSMA-11 PET for pelvic lymph nodes and distant metastasis. Recently, a randomized prospective trial showed that CI underestimates the number of patients with distant disease [5]. Looking in-depth into the data of this trial, 22 of 150 patients were suspected to have distant disease, with 11 false-negative cases by CI. Assuming that 128 patients would have been undergoing RPE, in these 11 cases, PSA persistence would be expected (8.6%); interestingly, this number corresponds well with the 8.8% of cases with PSA persistence despite R0 resection in our CI group. For the PSMA group, this result is significantly better in the prospective trial, with 23 of 145 patients with suspected distant disease and only two false-negative cases and, therefore, an expected PSA persistence rate after R0 RPE of 1.6% for 122 PSMA-PET negative cases. Again, this number is corresponding well to the PSA persistence despite R0 resection in our PSMA-group of 2.4%.

The superior rate of R0 margins in cases of unilateral nerve-sparing resections further supports the so far retrospectively collected data that shows PSMA-PET can improve local staging and guide the surgeon in the selection of appropriate safety margins (Fig. 4).

Subgroup analysis with inclusion of only high-risk patients accentuated the difference of outcome between the CI and PSMA groups, with the latter achieving a significantly lower rate of PSA persistence after RPE (*p* = 0.04) despite the relatively small cohort size. This is in accordance with our recently published study that showed PSMA-PET has a higher impact on management on high-risk patients [13].

**Fig. 6:**
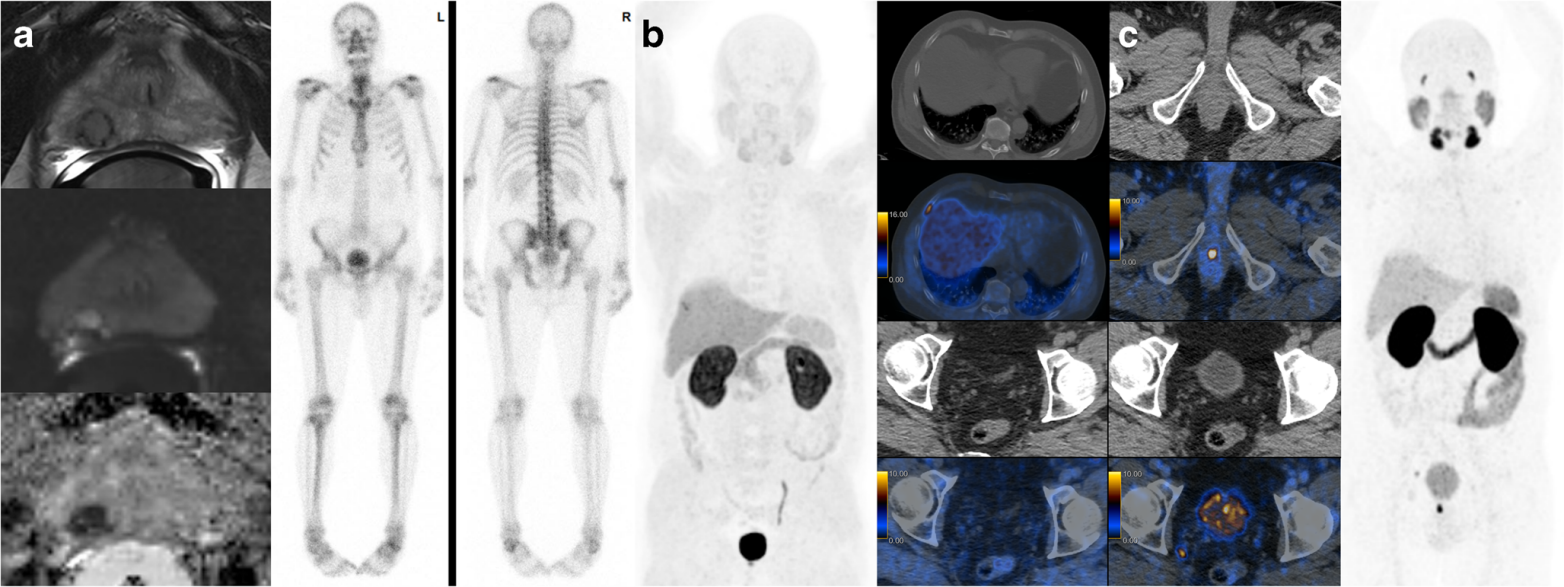
^68^Ga-PSMA-11 positron emission tomography/magnetic resonance imaging (^68^Ga-PSMA-11 PET/MRI) depicts lesions missed by choline PET. **(A)** Staging prostate MRI (top T2-weighted fast recovery fast spin-echo sequence, middle diffusion-weighted sequence, and bottom apparent diffusion coefficient) and bone scan of a 64-year-old patient with a T1c, Gleason score 4 + 4 = 8 tumor, and initial PSA level of 14.3 ng/ml show a PIRADS 4 lesion without suspicious pelvic lymph nodes nor bone metastasis. The patient underwent radical prostatectomy and had a persistent PSA value of 5.27 ng/ml 7 weeks after the surgery. **(B)** Subsequent choline PET showed a single suspicious rip lesion that was then treated with radiotherapy causing only a slightly drop of the PSA to 4.2 ng/ml with a subsequent rise to 7.15 ng/ml. **(C)** Following the PSA rise, ^68^Ga-PSMA-11 PET/CT was performed and showed lymph node metastasis and local recurrence

Of course, also PSMA-PET can miss distant disease, as in our cohort one patient with a strongly PSMA-positive primary tumor and one PSMA-positive internal iliac node had PSA persistence of 0.82 ng/ml despite RPE with R0 resection and nodal dissection with only one positive internal iliac node lymph node. He underwent salvage radiotherapy including the lymphatic drainage that reduced the PSA to 0.3 ng/ml (Fig. 5). On the other hand, there were three patients with PSA persistence despite R0 resection in our CI group, with PSA values of 0.33 ng/ml, 5.27 ng/ml, and 11.9 ng/ml. For the first patient, salvage radiotherapy was performed 6 months after surgery. The second patient had subsequent PSMA-PET imaging showing distant disease (Fig. 6), and for the third patient, with a PSA of 11.9 ng/ml, nodal-positive disease, and a negative choline PET, ADT was started.

The retrospective nature for outcome analysis is always a limiting factor, especially when analyzing postsurgical outcomes, given that there are many factors affecting RPE outcome. Preoperative mpMRI affects the rate of positive surgical margins in patients with clinical T1c disease. MRI-targeted biopsy, together with the number of systematic cores, influences histopathological classification of tumors [21, 22]. We therefore selected patients from one center, which underwent mpMRI, MRI-guided and saturation template biopsy followed by robotic surgery with pelvic lymph node dissection within 4.5 years. Even though most patients staged with PSMA-PET were operated in more recent years compared with the CI group, the fact that we focused only on the immediate post-surgical outcome and that the therapeutic approach did not change in our department within this time frame should reduce a potential bias in favor to PSMA-PET. Another limitation of our non-randomized retrospective design is the concern of unequal risk distribution between both groups. Indeed, due to the fact that PSMA-PET is used rather in high-risk patients, the PSMA group had a significantly higher risk before surgery compared with the CI group. However, despite a significantly higher risk profile, the PSMA group had a lower PSA persistence rate compared with the CI group.

The heterogeneous staging in the CI group including choline PET in 45% of the patients is also a limitation. However, the exclusion of the patients who underwent choline PET before surgery would have substantially reduced our CI group and further increased the bias for a higher risk in the PSMA group, given that 75% of the high-risk patients in the CI group underwent choline PET. Choline was not widely established for staging PCa; nevertheless, there is a number of publications that show a slight superiority or at least non-inferiority of choline PET compared with CI [23]. We therefore included patients that had choline PET before RPE for our CI group.

The higher risk profile of the PSMA-PET group as well as the inclusion of patients who underwent choline PET in the CI group might have underestimated the potential of PSMA-PET to improve patient’s oncological outcome in our study. Therefore, our finding of a better outcome in the PSMA group warrants further prospective studies to assess the impact of PSMA-PET on patient outcome and overall survival.

## Conclusion

^68^Ga-PSMA-11 PET has the potential to improve immediate RPE outcome, especially in patients with high-risk disease, where we showed that patients in the PSMA group had a significantly lower rate of PSA persistence after RPE compared with patients that underwent CI. Looking into all consecutively operated patients, there was a significant shift of RPE towards higher risk patients when PSMA-PET was available, without an increase in R1 resections or PSA persistence.

## Data Availability

Patient imaging was done in the scope of the routine clinical diagnostic studies, and the raw data are stored in the hospital archiving system at the Zurich University Hospital, Zurich, Switzerland.

## List of abbreviations

CI: conventional imaging
IQR: interquartile range
MRI: magnetic resonance imaging
mpMRI: multiparametric magnetic resonance imaging
pLND: pelvic nodal dissection
PET: positron emission tomography
PCa: prostate cancer
PSMA: prostate-specific membrane antigen
RPE: radical prostatectomy
